# HTLV-1 Tax mutants that do not induce G_1 _arrest are disabled in activating the anaphase promoting complex

**DOI:** 10.1186/1742-4690-4-35

**Published:** 2007-05-29

**Authors:** Randall Merling, Chunhua Chen, Sohee Hong, Ling Zhang, Meihong Liu, Yu-Liang Kuo, Chou-Zen Giam

**Affiliations:** 1 Department of Microbiology and Immunology, Uniformed Services University of the Health Sciences, 4301 Jones Bridge Rd., Bethesda, MD 20814, USA

## Abstract

HTLV-1 Tax is a potent activator of viral transcription and NF-κB. Recent data indicate that Tax activates the anaphase promoting complex/cyclosome (APC/C) ahead of schedule, causing premature degradation of cyclin A, cyclin B1, securin, and Skp2. Premature loss of these mitotic regulators is accompanied by mitotic aberrations and leads to rapid senescence and cell cycle arrest in HeLa and *S. cerevisiae *cells. Tax-induced rapid senescence (*tax*-IRS) of HeLa cells is mediated primarily by a dramatic stabilization of p27^*KIP *^and is also accompanied by a great surge in the level of p21^*CIP1*^mRNA and protein. Deficiencies in p27^*KIP *^prevent Tax-IRS. A collection of *tax *point mutants that permit normal growth of *S. cerevisiae *have been isolated. Like wild-type *tax*, many of them (C23W, A108T, L159F, and L235F) transactivate both the HTLV-LTR and the NF-κB reporters. One of them, V19M, preferentially activates NF-κB, but is attenuated for LTR activation. None of the mutants significantly elevated the levels of p21^*CIP1*^and p27^*KIP1*^, indicating that the dramatic surge in p21^*CIP1*/*WAF1*^and p27^*KIP 1*^induced by Tax is brought about by a mechanism distinct from NF-κB or LTR activation. Importantly, the ability of these mutants to activate APC/C is attenuated or abrogated. These data indicate that Tax-induced rapid senescence is causally associated with APC/C activation.

## Background

Human T-lymphotropic virus type I (HTLV-I) is the etiologic agent of adult T-cell leukemia and lymphoma, which occurs in approximately 5% of infected individuals after a long latency period lasting up to 20–40 years. The HTLV-1 viral transactivator/onco-protein Tax is thought to play an important role in T-cell malignancy and HAM/TSP. Tax transactivates the HTLV-1 LTR promoter through its interaction with CREB/ATF-1 [1-6], CBP/p300 [7-11], and the Tax-responsive 21-bp repeat element, and activates the NF-κB pathway [12-17] through the interaction with PP2A/IKKγ [18]. In addition to its transactivation functions, Tax also impacts on many aspects of the cell cycle: activating G_1_/S transition [19-21], inactivating p53 functions [22], inducing p21^*CIP1*/*WAF1*^mRNA transcription [23-26], and inhibiting apoptosis and DNA repair [27,28]. Recent data have indicated that Tax can dramatically perturb mitotic regulation, causing micronuclei formation, cytokinesis failure, and chromosome instability [29,30]. ATL cells are often aneuploid with complex chromosomal abnormalities including trisomy 3, trisomy 7, a partial deletion of 6q, and abnormalities of 14q11 [31]. Large lymphocytes with cleaved/cerebriform nuclei are also frequently seen in HTLV-I-positive individuals [32-35]. These pathological findings are likely to be associated with Tax-induced mitotic aberrations.

Indeed, in *tax*-expressing HeLa, MT4, and *S. cerevisiae *cells, the levels of cyclin A, cyclin B and the anaphase inhibitor: securin/Pds1p (precocious dissociation of sister chromatids) were found to be significantly reduced [30]. We have found that the loss of cell cycle regulators and the mitotic defects induced by Tax may be causally linked and are associated with premature activation of the anaphase promoting complex/cyclosome (referred to as APC/C henceforth), a multiprotein E3-ubiquitin ligase that controls the onset of anaphase and mitotic exit by targeting mitotic cyclins and other cell cycle regulators for degradation [36]. More recently, we have shown that the cell cycle dysregulation induced by *tax *does not end with mitotic abnormalities. *Tax*-transduced HeLa cells, after passage through a faulty cell division cycle, immediately entered into a senescence-like G_1 _arrest termed *tax*-induced rapid senescence, *tax*-IRS [37]. These cells expressed high levels of Cdk2 inhibitors: p21^*CIP1*/*WAF1*^and p27^*KIP 1*^as a consequence of Tax-mediated activation of p21^*CIP1*/*WAF1*^mRNA transcription, and increased stabilization of p21^*CIP1*/*WAF1*^and p27^*KIP 1*^proteins. Consistent with these findings, Tripp et al have also reported that expression of *tax *can cause CD34+ hematopoietic cells to cease proliferation [38].

During normal cell cycle progression, p21^*CIP1*/*WAF1*^and p27^*KIP 1*^transiently accumulate during G_1_, but become degraded in S. The destruction of p21^*CIP1*/*WAF1*^and p27^*KIP 1*^during S phase is regulated by the multisubunit E3 ubiquitin ligase, SCF (Skp-Cullin-F box), together with its substrate-targeting subunit, Skp2 [39-44] and the cell cycle regulatory protein, Cks1 [39,44,45]. Recent evidence indicates that Skp2 and Cks1 are both substrates of the Cdh1-associated APC/C (APC^Cdh1^). They become polyubiquitinated and degraded in late M and early G_1 _when APC^Cdh1 ^is highly active. This renders SCF^SKP2 ^inactive and allows p21^*CIP1*/*WAF1*^and p27^*KIP 1*^levels to build up in G_1_. When *tax *is expressed, APC/C becomes prematurely activated. This causes Skp2 to be polyubiquitinated and degraded starting in S, through G_2_/M and in subsequent G_1_. The drastic reduction in Skp2 and possibly Cks1, apparently inactivated SCF^SKP2^, profoundly stabilized p21^*CIP1*/*WAF1*^and p27^*KIP1*^, thereby committing cells to senescence. The stabilization and surge of p21^*CIP1*/*WAF1*^and p27^*KIP 1*^in *tax*-expressing cells, therefore, is temporally and causally linked to premature APC/C activation. In essence, Tax activates the cellular program for mitotic exit far ahead of schedule, thereby driving cells into a state of permanent arrest. Interestingly and as might be predicted, we have found that HTLV-1 transformed T-cells invariably express lower levels of p27^*KIP1*^. Indeed, a loss of p27^*KIP 1*^function allows cells to evade *tax*-IRS [37].

Our earlier results have indicated that expression of Tax in *S. cerevisiae *also leads to unscheduled, APC-mediated degradation of Clb2p and Pds1p, G_2_/M delay, chromosome aneuploidy, growth arrest, and loss of cell viability [30]. Considering the highly conserved nature of the cellular machineries that control mitosis in eukaryotes, this is probably not surprising. The powerful genetics available for *S. cerevisiae *provides an opportunity to dissect the mechanism by which Tax dysregulates APC/C and mitosis, which is otherwise difficult to perform in human cells. Here we report the isolation of a collection of 26 *tax *point mutants whose expression in *S. cerevisiae *did not lead to growth arrest. Five mutants (V19M, C23W, A108T, L159F, and L235F) – with amino acid substitutions that span the majority of Tax protein sequence – were chosen for in-depth analyses. C23W, A108T, L159F, and L235F transactivated both the HTLV-LTR and the NF-κB reporters. One mutant, V19M, preferentially activated NF-κB, but was attenuated in LTR activation. All became impaired or abrogated in their ability (i) to activate APC, (ii) to increase the levels of p21^*CIP1*/*WAF1*^and p27^*KIP1*^, and (iii) to cause *tax*-IRS. These data strongly suggest that *tax*-IRS, with the associated mitotic aberrations and the accompanying rise in p21^***CIP1*/*WAF1 ***^and p27^*KIP 1*^levels, is coupled to APC/C activation, and is mechanistically unrelated to the CREB/ATF-CBP/p300 or IKK-NF-κB pathway.

## Results

### Isolation of *tax *mutants that do not cause growth arrest in *S. cerevisiae*

**Figure 1:**
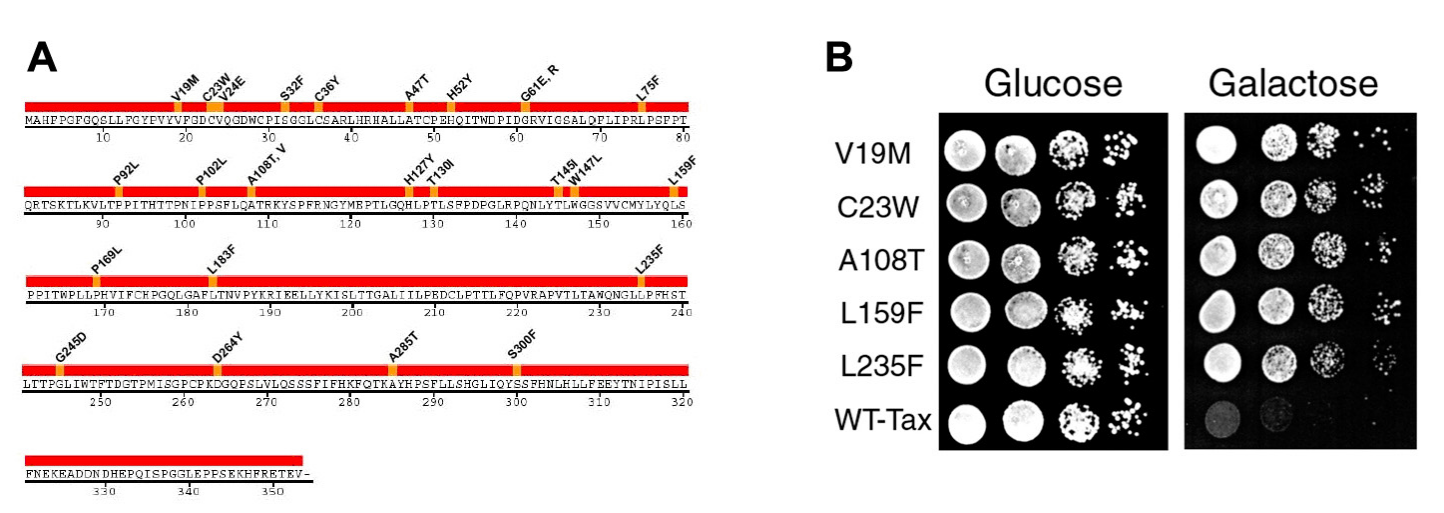
**(A) A summary of amino acid substitutions in HTLV-1 *tax *point mutants whose expression did not cause growth arrest in *S. cerevisiae***. The complete amino acid sequence of HTLV-1 Tax is shown with the amino acid alteration in each point mutant indicated above. **(B) Expression of 5 representative HTLV- *tax *point mutants in *S. cerevisiae***. W303-1a cells were transformed with the Gal10-Tax (WT-Tax) or its equivalent carrying each of five mutant *tax *alleles and plated on agar plates containing 2% raffinose or 2% raffinose plus 2% galactose. The amino acid alterations in *tax *mutants are as indicated.

In the course of a yeast 2-hybrid screen using Tax as bait, we noticed that the yeast strain expressing the *lexA-tax *fusion grew significantly slower than the *lexA *control. This prompted us to examine more closely the effect Tax exerts on the growth and proliferation of *S. cerevisiae*. To this end, W303a, a standard laboratory yeast strain, was transformed with pRS315-Gal10-Tax, a CEN plasmid carrying the *tax *gene under the control of a galactose inducible promoter [30]. As reported previously [30], expression of *tax *after galactose induction lead to a cessation of cell growth and proliferation. Upon Tax expression, the W303a/Gal10-Tax cells initially suffered a delay in S/G_2_/M progression [30]. They then became arrested at G_1 _phase of the cell cycle. The growth-arrested cells became greatly enlarged in size, but were without buds and displayed severe DNA aneuploidy [30]. Their viability was also significantly decreased. These results immediately suggest that *tax *mutants that do not cause growth and proliferation arrest may be readily isolated in *S. cerevisiae *and these mutants may have similar or identical properties in human cells. To isolate *tax *mutants impaired in causing growth arrest, we mutated pRS315-Gal10-Tax by hydroxylamine (36). W303a cells were then transformed with the pool of chemically mutated plasmid preparation, and plated to select for galactose-resistant transformants. The colonies on galactose plates were then screened by colony dot blots for *tax *expression using a mouse hybridoma Tax antibody, 4C5. A total of 26 non-growth-arrest *tax *mutants were identified, for which the respective pRS315 vectors were isolated. We next confirmed loss of the growth arrest phenotype by reintroducing plasmids carrying the mutant *tax *alleles into W303a. As expected, all W303a expressing mutant *tax *alleles readily grew on both glucose and galactose plates (Fig. 1B), while W303a expressing the wild-type *tax *failed to grow on galactose plates as previously described [30].

DNA sequence analyses of the *tax *coding sequence revealed that each of the 26 *tax *mutants contained a single amino acid substitution that resulted from a G to A or C to T transition, as might be expected for hydroxylamine mutagenesis. The altered amino acid residues in the Tax protein sequence are listed in Fig. 1A. Many of the amino acid substitutions are clustered in the NH_2_-terminal half of Tax (20/26). Consistent with the notion that the amino acid substitutions had occurred in important regions of Tax, we noticed that the T130I substitution overlap with the dual amino acid substitutions – T130A L131S – in a well characterized *tax *mutant known as M22, which is partially defective in dimerization and is severely impaired in IKKγ/NEMO-binding and NF-κB activation. Two distinct mutations (G61E and G61R) and (A108T and A108V) were isolated for each of the amino acid residues 61 and 108, suggesting the importance of these residues in protein-protein interactions that mediate Tax functions. Finally, the expression levels of all mutants in *S. cerevisiae *were comparable as judged by immunoblotting (data not shown).

### Tax mutants selected in W303a are functional in HTLV-1 LTR and NF-κB trans-activation

**Figure 2:**
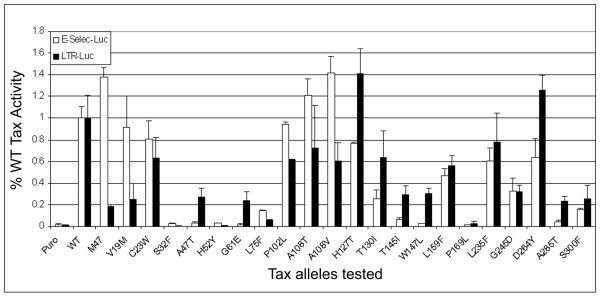
**HTLV LTR and NF-κB transactivation functions of *tax *mutants**. The mutant *tax *alleles were subcloned into a lentivirus vector, HR'CMV-SV-puro. The activity of each mutant to transactivate HTLV-1 LTR and NF-κB was determined by cotransfection of an HTLV-1 LTR luciferase construct or an E-selectin luciferase construct with each HR'CMV-*tax *mutant construct into 293T cells. The HTLV LTR (solid bars) and NF-κB (open bars) reporter activities of each mutant were normalized against those of the wild-type *tax *and expressed as % wild-type activity.

**Figure 3:**
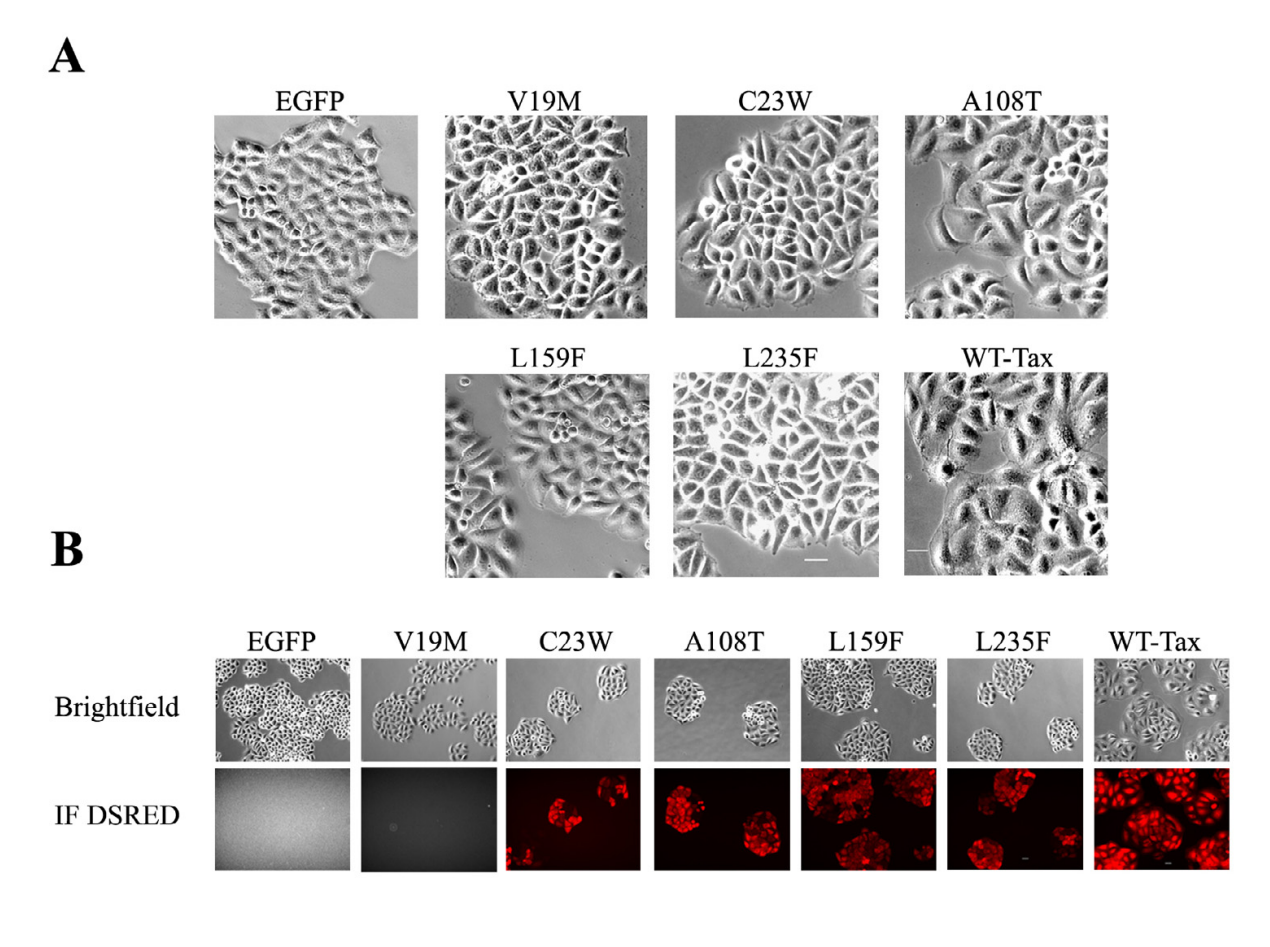
**(A) A comparison of the morphologies of HeLa cells transduced with *tax *mutants**. Morphology of Hela cells transduced with a lentivirus vector carrying, respectively, the wild-type, V19M, C23W, A108T, L159F, and L235F mutant *tax *alleles. The *tax*-transduced cells were selected in medium containing puromycin 1 μg/ml for 3 days. The puromycin-resistant colonies were photographed. The scale bar represents 20 μm. **(B) Transactivation of a Tax-specific reporter HeLa cell line transduced with *tax *mutants**. A HeLa cell line containing a stably integrated Tax reporter cassette, 18 × 21-DsRed, was transduced with a lentivirus vector, HR'CMV-SV-puro, harboring wild-type or each of the mutant tax alleles. The *tax*-transduced cells were then selected in medium as above. Puromycin-resistant colonies were visualized and photographed using an Olympus IX8 inverted fluorescence microscope. The scale bar represents 20 μm.

Next, we investigated the biological activities of *tax *mutants in mammalian cells. Mutant *tax *alleles were cloned into a lentiviral vector, HR'CMV-SV40-puro. This vector allowed *tax *to be expressed transiently from the CMV immediate early promoter after DNA transfection or stably after lentivirus vector-mediated gene transduction. We first examined the ability of the Tax mutants to transcriptionally activate luciferase reporters driven respectively by the HTLV-1-LTR (LTR-Luc) and the NF-κB-inducible E-selectin-promoter (E-selec-Luc) [46]. Twenty one mutants were analyzed by luciferase reporter assays (Fig. 2). The other five mutants (V24E, C36Y, G61R, P92L, and L183F) were excluded from the reporter assays because of either the drastic amino acid alterations caused by the mutations or the existence of alternative amino acid substitution in the same position. Approximately half of the mutants analyzed (S32F, A47T, H52Y, G61E, L75F, T145I, W147L, P169L, A285T, and S300F) were greatly impaired in both transactivation functions of Tax. Many of these mutations are in the highly conserved NH_2_-terminus of Tax. Because of their severe defects, no attempts have been made to determine if their expression in 293T cells may be altered by the respective amino acid substitutions. The levels of expression of these mutants in *S. cerevisiae *were normal, however. By contrast, several mutants (C23W, P102L, A108T, A108V, H127T, L159F, 235F, G245D, and D264Y) continued to transactivate both LTR and NF-κB reporters to levels (greater than 50%) comparable to those of the wild-type Tax. Of note, V19M was specifically impaired in LTR activation but remained a potent NF-κB activator, while T130I was defective in NF-κB activation, but exhibited significant LTR activation capability, reminiscent of similar properties of the M22 (T130A L131S) mutation mentioned above. As indicated below, at least five of these mutants (V19M, C23W, A108T, L159F, and L235F) expressed at levels comparable to that of the wild-type Tax in HeLa cells (see below). These results indicate that mutations that impaired the ability of Tax to arrest growth of W303a cells did not necessarily affect LTR or NF-κB transactivation. Finally, although the reporter assays for some of the mutants varied more than others, the variations occurred mostly due to strong transactivation; and importantly, the LTR and NF-κB transactivations by the 5 mutants that were analyzed in depth have been confirmed by independent methods (Fig. 3 and see below). We infer from these data that the growth arrest phenotype of Tax most likely involves interactions with a cellular process distinct from the CREB/CBP/p300 and the IKK/NF-κB pathways.

### Expression of *tax *mutants in HeLa cells

We next selected 5 mutants (V19M, C23W, A108T, L159F, and L235F) that retained the ability to transactivate LTR and/or NF-κB for further analysis. Lentivirus vectors (LV) capable of transducing the mutant *tax *alleles were generated by co-transfection of the respective HR'-CMV-*tax*-SV40-puro vectors together with packaging plasmids that encode HIV structural proteins and VSV G protein as previously reported [37,47]. A stable HeLa cell line, HeLa-18 × 21-DsRed, which expresses DsRed under the control of a Tax-inducible enhancer/promoter cassette containing 18 copies of the 21-bp repeat upstream of a minimal HTLV-1 promoter [48], was used as the cellular background for introducing the *tax *alleles. As the expression of DsRed in HeLa-18 × 21-DsRed is strictly Tax-dependent, cells that express Tax after gene transduction can be readily detected by fluorescence microscopy (Fig. 3). HeLa-18 × 21-DsRed cells were infected with LV carrying the wild-type, V19M, C23W, A108T, L159F, L235F *tax *alleles, or the EGFP gene. The LV-transduced cells were then selected in media containing 1 μg/ml puromycin for 2–3 days. Drug-resistant colonies were then grown in puromycin-free medium for 1 day and observed under a fluorescence microscope for DsRed expression. In agreement with the LTR-Luc reporter activities described above (Fig. 2), C23W, A108T, L159F, and L235F, but not V19M activated DsRed expression (Fig. 3B). As expected, HeLa-18 × 21-DsRed transduced with the LV-EGFP control did not express DsRed. Previously, we have demonstrated that Tax expression in HeLa cells greatly elevated the levels of p21^*CIP1*/*WAF1*^and p27^*KIP 1*^cyclin-dependent kinase inhibitors, thereby causing HeLa cells to enter into a senescence-like G_1 _arrest termed Tax-induced rapid senescence (Tax-IRS) [37]. The HeLa cells in Tax-IRS are flat, enlarged, vacuolated, often binucleated, and stained positive for the senescence associated β-galactosidase. Indeed, in agreement with previous results, microscopic examination of the HeLa-18 × 21-DsRed cell line transduced with LV-Tax (wild-type) revealed a prevalence of enlarged and binucleated cells, consistent with the notion that they were in the state of Tax-IRS (Fig. 3A). By contrast, the morphology of cells transduced with mutant *tax *alleles, with the exception of A108T, resembled those of control cells transduced with the EGFP gene. Finally, we noted that despite some similarity of A108T cells to Tax (wild-type) cells, the extent of arrest and morphological changes in A108T cells appeared to be attenuated (Fig. 3A).

### Tax mutants whose expression is permissible in *S. cerevisiae *do not cause, or are attenuated in inducing cell cycle arrest in HeLa cells, but remain functional in activating I-κB degradation and p100 processing

**Figure 4:**
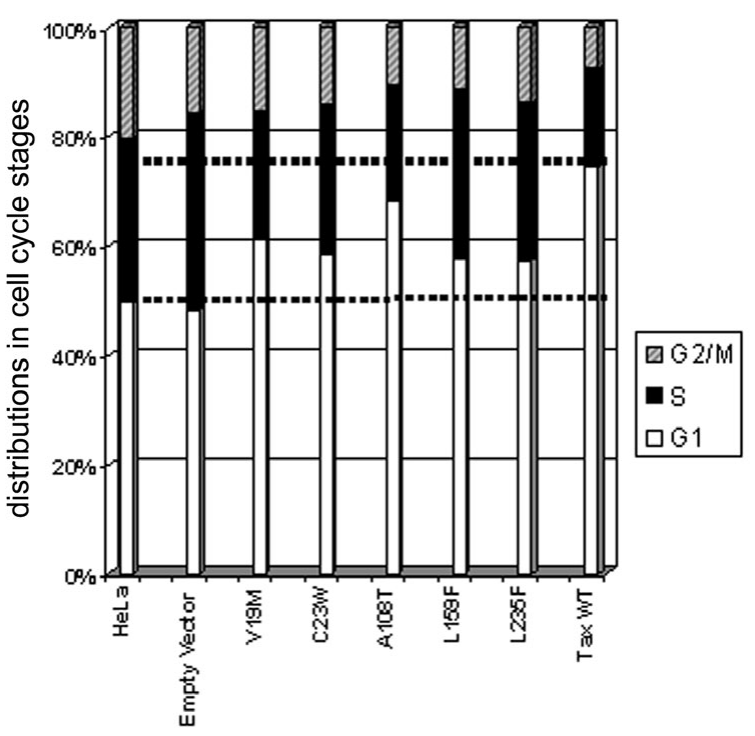
***S. cerevisiae*-viable *tax *mutants are attenuated in inducing cell cycle arrest/senescence in HeLa cells**. HeLa cells transduced with a lentivirus vector carrying wild-type or mutant *tax *were selected in puromycin for 72 h as above and then grown in puromycin-free medium for 24 h. Cells in each group were then fixed in 70% ethanol, stained with propidium iodide, and analyzed by flow cytometry. (A) Flow cytometry chromatograms. (B) A bar graph representation of the fraction of cells in G_1_, S, G_2_/M phases of the cell cycle after transduction with *tax *alleles.

To characterize the various *tax *mutants further, we analyzed the LV-mutant-*tax*-transduced cells by flow cytometry. Three days after puromycin selection, asynchronously grown LV-transduced cells were transferred to puromycin-free medium for 24 h and harvested for analyses. As anticipated from the cell morphology in Fig. 3A, most cells that expressed the wild-type Tax (75%) appeared in the G_1 _phase of the cell cycle (Fig. 4). In contrast, G_1 _populations for cells transduced with the various *tax *mutant alleles were significantly lower, albeit somewhat higher than that of the EGFP-transduced control (Fig. 4). These results support the notion that those *tax *mutants that failed to cause growth arrest in *S. cerevisiae *are also significantly disabled or attenuated in inducing senescence/cell cycle arrest in mammalian cells, albeit with varying degrees of attenuation.

**Figure 5:**
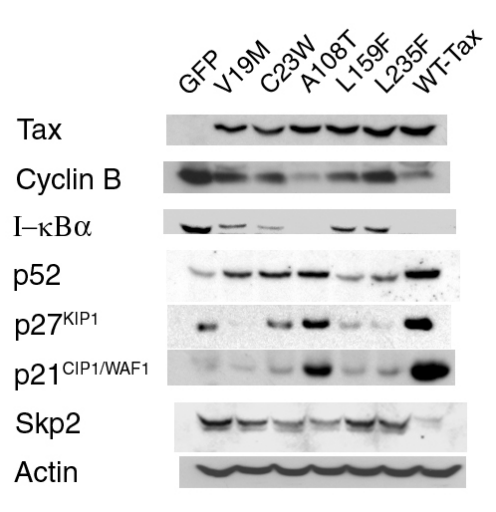
**Immunoblot analyses of HeLa cells transduced with wild-type or mutant *tax *alleles**. HeLa cells transduced with the wild-type or the respective mutant *tax *allele were harvested. Cell lysates were prepared, resolved by SDS-12% PAGE, and probed with antibodies against Tax, cyclin B1, I-κBα, NF-κB, p52, p27^*KIP1*^, p21^***CIP1*/*WAF1***^, Skp2, and actin, respectively.

The phenotypes of the *tax *mutants were not due to variations in the levels of Tax protein expression as indicated by immunoblotting (Fig. 5). In accordance with the overall cell morphology and flow cytometry analyses, the levels of p21^*CIP1*/*WAF1*^and p27^*KIP 1*^in the various *tax*-transduced cells correlated with their extent of growth arrest or lack thereof, with wild-type *tax *greatly increasing the levels of p21^*CIP1*/*WAF1*^and p27^*KIP1*^, followed by the A108T mutant, and with the remaining mutants having only moderate to no effect (C23W, L159F, L235F, V19M). As might be expected, the levels of cyclin B1 in the respective cell lines inversely correlated with the growth characteristics of the respective cells. Likewise, the levels of Skp2 in the transduced cells also correlated with their respective cyclin B1 levels. Finally, consistent with the ability of the *tax *mutants to transactivate the E-seletin-Luc NF-κB reporter (Fig. 2), the levels of I-κBα in the *tax*-transduced cells were reduced, while those of p52, the mature NF-κB2, were increased. Here again, in general agreement with the reporter assays, the A108T mutant is equivalent or possibly better than the wild-type *tax *in inducing I-κBα degradation and p52 NF-κB processing, second by V19M and C23W, followed lastly by L235F and L159 mutants. While for some mutants (A108T, L159F, and L235F) there appears to be some correlation between the severity of cell cycle arrest/senescence phenotype and the degree of NF-κB activation, for others such as V19M and C23W, that are strong NF-κB activators, the senescence phenotype was significantly attenuated. These results support the notion that the Tax-induced cell cycle arrest/rapid senescence (Tax-IRS) and increase in p21^*CIP1*/*WAF1*^and p27^*KIP 1*^levels are causally related and do not involve directly either the CREB/CBP/p300 or the IKK/NF-κB pathway. Whether the IKK/NF-κB pathway may share a common Tax-targeted regulatory factor with the cell cycle/APC pathway remains to be seen.

### *S. cerevisiae*-viable *tax *mutants are attenuated in APC/C activation

**Figure 6:**
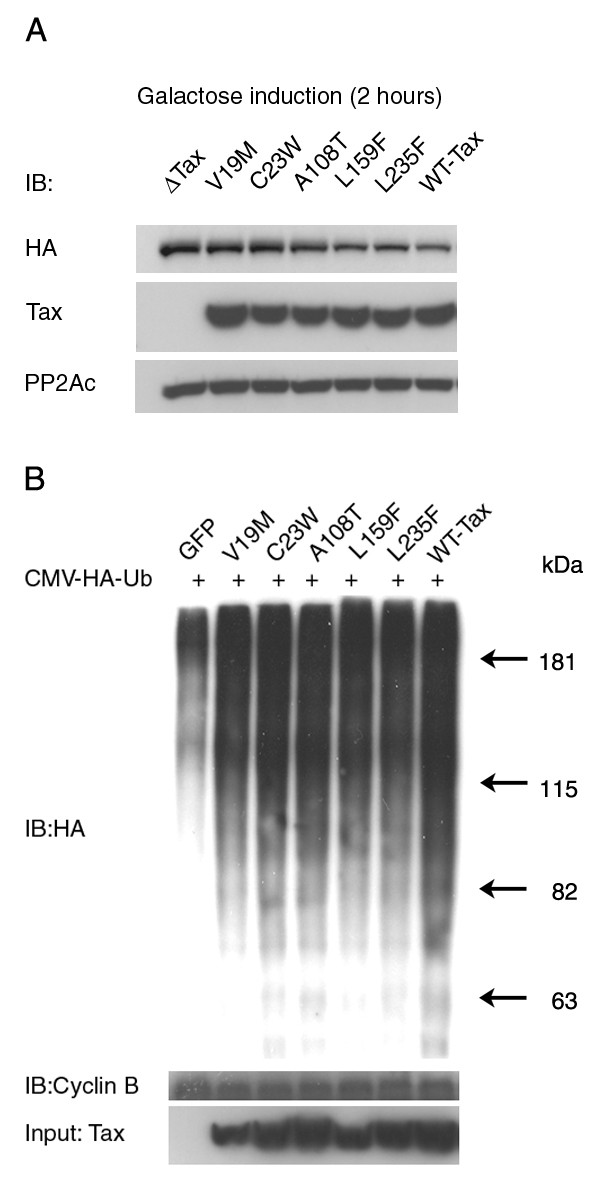
***S. cerevisiae*-viable *tax *mutants are attenuated in APC/C activation**. (A) KY630 cells carrying a gene encoding CLB2-3XHA integrated at the CLB2 locus were transformed with either Gal10-wild-type tax or Gal10-mutant *tax*. KY630/Gal10-*tax *cells were cultured in SC medium containing 2% raffinose at 30°C overnight for 12 h, then diluted to 0.3 A_600_, grown to mid-log phase, and induced for *tax *expression by the addition of 2% galactose. Cell lysates were prepared after 120 minutes. Immunoblots were carried out with anti-HA and 4C5 monoclonal antibodies. (B) 293T cells were transfected with a plasmid encoding HA-tagged human ubiquitin together with an expression plasmid for wild-type tax or mutant *tax*. The cells were arrested at the G_1_/S border by a single thymidine treatment, released into complete DMEM containing 10 μM MG132 for 5 h, immunoprecipitated with cyclin B1 antibody and immunoblotted with an HA antibody to detect polyubiquitinated cyclin B1.

We have shown previously that the mitotic abnormalities and rapid senescence that Tax induces in *S. cerevisiae *and HeLa cells are associated with unscheduled activation of the anaphase promoting complex and the premature degradation of mitotic/cell cycle regulators including cyclin A, Clb2/cyclin B, Pds1/securin, and Skp2 [30]. The levels of cyclin B1, Skp2, p21^*CIP1*/*WAF1*^and p27^*KIP 1*^in the HeLa cells expressing the various *tax *alleles suggest that the *S. cerevisiae*-viable *tax *mutants are impaired in APC/C activation. To determine the effect of the *tax *mutants on APC/C directly, we introduced them into a yeast strain, KY630, which contains a chromosomally integrated HA-CLB2 at the CLB2 locus. As anticipated, upon induction of *tax *expression for 2 h, a reduction of Clb2p in cells expressing wild-type *tax *was observed compared to the *tax*-null control. By contrast, the *S. cerevisiae*-viable V19M, C23W, A108T, L159F, and L235F *tax *mutants were attenuated in causing Clb2p reduction/degradation (Fig. 6A). We have shown previously that Tax activates the anaphase promoting complex in *S. cerevisiae*, HeLa, 293T and HTLV-1 transformed T cells [36]. For the ease of transfection, the wild-type and mutant *tax *alleles were individually co-transfected with HA-tagged ubiquitin into 293T cells. Indeed, the five *tax *mutants were also found to be attenuated in inducing cyclin B1 polyubiquitination when compared to the wild-type control (Fig. 6B). The extents of attenuation of the five *tax *mutants in *S. crevevisiae *versus 293T cells were not exactly identical (compare Fig. 6A and 6B). This may reflect subtle structural differences between *S. cerevisiae *and human anaphase promoting complex. Finally, the degrees of the mutants to cause cyclin B1 polyubiquitination (A108T ≥ C23W > L159F, L235F, and V19M) correlated largely with the levels of p21^*CIP1*/*WAF1*^and p27^*KIP 1*^increase in the transduced cells (A108T > C23W > L159F, L235F, and V19M). Taken together, these results support the idea that unscheduled activation of the anaphase promoting complex is responsible for the Tax-induced rapid senescence/cessation of cell proliferation in both human and *S. cerevisiae *cells.

## Discussion

In this study, we have described a collection of 26 *tax *single-point mutants that are disabled in causing cell cycle arrest in *S. cerevisiae*. A total of 21 *tax *alleles were analyzed further by luciferase reporter assays for LTR and NF-κB transactivation. Approximately half of the amino acid substitutions likely have impacted on critical regions of Tax so as to render it severely defective. Special attention was directed to five mutants (V19M, C23W, A108T, L159F, and L235F) that remained strong LTR and NF-κB transactivators. Their phenotypes in HeLa cells were largely consistent with those seen in *S. cerevisiae *– all were attenuated or significantly impaired in causing p21^*CIP1*/*WAF1*^and p27^*KIP 1*^accumulation, but remained able to induce I-κB degradation and p100 NF-κB2 processing. Whereas the majority of HeLa cells transduced with wild-type *tax *entered into *tax*-IRS, cells transduced with each of the 5 mutant *tax *alleles continued to proliferate, albeit at rates that varied dependent on the levels of p21^*CIP1*/*WAF1*^and p27^*KIP 1*^expressed. These results validated the utility of *S. cerevisiae *as a model for dissecting the mitotic abnormalities and rapid senescence/cell cycle arrest induced by Tax.

The levels of p21^*CIP1*/*WAF1*^and p27^*KIP 1*^are regulated through transcription, phosphorylation (by cyclinE/Cdk2), subcellular localization, ubiquitination, and proteasome-mediated degradation [40,49,50]. The E3 ubiquitin ligase, SCF, together with its substrate-recognition subunit, Skp2, mediates the ubiquitination and degradation of p21^*CIP1*/*WAF1*^and p27^*KIP 1*^[39,44,45]. The level of Skp2 oscillates in a cell cycle-dependent manner. Recent data have indicated that Skp2 and another SCF subunit, Cks1, are substrates of the Cdh1-associated APC/C (APC^Cdh1^) [39,44]. Both become ubiquitinated and degraded in late M and early G_1 _when APC^Cdh1 ^is highly active. This inactivates SCF and allows p21^*CIP1*/*WAF1*^and p27^*KIP 1*^to accumulate transiently in G_1_. We have shown recently that in HeLa cells transduced with *tax*, early APC/C activation sets in motion premature loss of cyclin A, cyclin B, securin, and Skp2, and causes a dramatic build-up of p21^*CIP1*/*WAF1*^and p27^*KIP 1*^during S phase. After an aberrant mitotic division cycle, the great surge in p21^*CIP1*/*WAF1*^and p27^*KIP 1*^in *tax*-expressing cells then commits cells into a state of irreversible cell cycle arrest. Results from the present analysis, i.e. mutations in *tax *that abrogated induction/stabilization of p21^*CIP1*/*WAF1*^and p27^*KIP 1*^also disabled APC/C activation, are in agreement with that conclusion.

Activation of p21^*CIP1*/*WAF1*^mRNA transcription by Tax has been reported previously [23,51-53]. Because a Tax mutant, M47, which is deficient in LTR activation, became disabled in activating p21^*CIP1*/*WAF1*^promoter, some of these earlier studies have suggested that Tax-induced increase in p21^*CIP1*/*WAF1*^resulted from transactivation via the CREB/ATF-CBP/p300 pathway [23,53]. Since four of the five mutants analyzed here activated LTR-luciferase reporter at levels (70–80%) comparable to that of the wild-type *tax*, yet were substantially impaired in causing p21^*CIP1*/*WAF1*^and p27^*KIP 1*^increase, we conclude that the CREB/ATF-CBP/p300 pathway is most likely not the principal determinant in the accumulation of p21^*CIP1*/*WAF1*^and p27^*KIP1*^. Likewise, several of the mutants – A108T, V19M and C23W, in particular – are potent activators of IKK-NF-κB as indicated by luciferase reporter assays and the extent of I-κB degradation and p100 processing. These mutants are nevertheless impaired in elevating p21^*CIP1*/*WAF1*^and p27^*KIP 1*^levels. These results support the notion that the NF-κB pathway is not directly responsible for the Tax-induced increase in p21^*CIP1*/*WAF1*^and p27^*KIP1*^. Our earlier data suggest that a major factor for Tax-induced p21^*CIP1*/*WAF1*^increase is correlated with premature APC/C activation [37]. Therefore, promoter transactivation by Tax may only contribute moderately to the overall build-up of p21^*CIP1*/*WAF1*^protein. This would explain the discrepancy between the data reported here and the earlier studies which relied heavily on p21^*CIP1*/*WAF1*^promoter-luciferase reporter assays. Finally, we have shown that via a tripartite interaction, Tax, PP2A and IKKγ form a stable ternary complex wherein PP2A activity is inhibited or diminished by Tax. In essence, PP2A inhibition by IKKγ-bound Tax maintains IKK in an active, phosphorylated state, causing constitutive phosphorylation and degradation of I-κB, which, in turn, leads to potent activation of genes under NF-κB/Rel control. Since PP2A regulates many critical cellular processes, it is conceivable that APC/C activation by Tax is also mediated through an inhibition of PP2A. In this sense, a *tax *mutant deficient in PP2A interaction will be disabled for both NF-κB and APC/C activation, but may continue to transactivate LTR. Several tax mutants, H43Q, K85N, and M22 (T130A, L131S) have been shown previously to be disabled for PP2A binding [18]. Both H43Q and M22 are deficient in NF-κB but not in LTR transactivation, while K85N is defective for both. Preliminary analyses suggest that these three mutants are also impaired in inducing cell cycle arrest. Mutants like H43Q and M22, with the possible exception of T130I, however, are not highly represented in the current collection. This may be due, in part, to the importance of the PP2A-binding domain of Tax in mediating other critical protein-protein interaction.

The biological/virological relevance for the profound cell cycle arrest induced by Tax remains unclear. It is possible that HTLV-1-infected T-cells that are arrested in a senescence-like state may persist longer *in vivo *and may, in this condition, be co-opted to devote significant cellular resources to virus replication. Alternatively, the dramatic morphological changes associated with the senescence-like arrest may facilitate virus assembly and/or transmission. Many of the *tax *mutants characterized here that remain functional in transactivating viral LTR and NF-κB can be incorporated into an infectious molecular clone of HTLV-1 to address this question.

## Methods

### Tax mutagenesis and selection for *tax *mutants

The CEN plasmid, pRS315 Gal10-Tax, that contains *tax *under the control of a galactose-inducible promoter derived from Gal10 gene has been previously described [30]. To introduce mutations into *tax *sequence, pRS315 Gal10-Tax plasmid DNA was exposed to hydroxylamine (1 mg/ml) overnight at 37°C [54]. The plasmid was then purified using a PCR purification kit (Qiagen), and used (1 μg) to transform *S. cerevisiae*. The transformants were plated on leucine-dropout plates that contained galactose as the sole carbon source. Colonies that appeared on galactose plates were then picked and seeded in grids on galactose plates, transferred to nitrocellulose filters, lysed, and screened for Tax expression using a monoclonal antibody (4C5) against Tax. Only positive clones were chosen for plasmid extraction/isolation. The plasmids extracted from W303-1a were than used to transform competent *E. coli *for DNA preparation and sequence analysis.

### Cloning mutant *tax *alleles into a lentiviral vector

The lentivirus vector, LV-Tax-SV-Puro, which contains the wild-type *tax *gene under the control of the CMV enhancer promoter and **the puromycin-resistance gene **expressed from the SV40 enhancer/promoter (SV-Puro), has been previously reported [37]. A mutant *tax *allele, M47, which carries a diagnostic BglII restriction site in the *tax *coding sequence, was cloned into LV-Tax-SV-Puro via the BamHI (located immediately upstream of the translational initiation codon) and SmaI (downstream of the M47 mutations) restriction sites to generate LV-M47-SV-Puro. Most mutant *tax *alleles were cloned into LV-M47-SV-puro similarly except that an internal MluI site and an XmaI site, located at the aforementioned SmaI site, were used. The recombinants were identified by a loss of the diagnostic BglII site from the recombinant, and confirmed by DNA sequence analysis. For NH_2_-terminal mutations that lie upstream of the MluI site, DNA fragments harboring the mutations were generated by PCR and cloned into LV-Tax-SV-puro via the BamHI and MluI sites. Primers used to amplify the NH_2 _terminal coding region of Tax are 5'TaxBamHI 5'-CGCGGATCCGCCACCATGGCCCACTTCCCAGGGTT-3' (with the translational start site underlined) and 3'TaxXmaI 5'-GCTCTAAGCCCCCGGGGGATA-3'.

### Construction of the Tax-inducible reporter, 18 × 21-DsRed and derivation of the 18 × 21-DsRed indicator cell line

A highly Tax-inducible enhancer/promoter cassette that contains 18 copies of the Tax responsive 21 bp repeat element upstream of a minimal HTLV-1 promoter (18 × 21) has been reported previously [48]. A blunt-ended BamHI fragment containing the 18 × 21 cassette was inserted upstream of the DsRed reporter gene in the pDsRed2-C1 (Stratagene) plasmid (blunt-ended at AseI and AgeI sites) to make p18 × 21DsRed-Neo. A HeLa reporter cell line for Tax was derived by transfecting cells with the p18 × 21-DsRed-Neo plasmid, followed by G418 (1 μg/ml, Invitrogen) selection. G418-resistant clones were expanded and transduced with the HR'CMV-Tax-puro lentiviral vector and observed for DsRed expression. One clone that had low basal expression but high DsRed expression in the presence of Tax was chosen for this study.

### DNA transfection and luciferase reporter assay

Approximately 10^5 ^293T cells/well in a 12-well plate were transfected with the expression construct for each of the *tax *alleles (0.5 μg) together with either HTLV- LTR-Luc (0.1 μg) or E-selectin-Luc reporter plasmid (0.5 μg) using a calcium phosphate transfection kit (Invitrogen). Forty eight hours after transfection, cells were lysed using 250 μl of reporter lysis butter and 20 μl lysate from each transfection was used for the luciferase assay. After injection of 100 ul luciferase substrate (Promega), the luciferase activity was measured by a MLX microtiter plate luminometer. Transactivation functions of V19M, C23W, A108T, L159F, and L235F mutants were further confirmed by including in the transfection mixture 0.5 μg of a control plasmid, pRL-TK, that contains the renilla luciferase reporter gene driven by the herpesvirus thymidine kinase promoter.

### Lentiviral vector production and gene transduction

Lentiviral vectors (LV) were produced as previously described after transfection of 293T cells [37]. Culture supernatants were harvested at 24, 48 and 72 h after transfection, pooled, filtered, aliquoted, and stored at -80 °C. Viral titers were measured by adding serially diluted LV stocks to 2 × 10^5 ^(HeLa 18 × 21-DsRed-Neo) cells were seeded in a 24-well plate. Polybrene (8 μg/ml, Sigma) was added to the medium together with the vector stocks to facilitate infection. Three days post-transduction, the number of RFP-positive cells in each well was counted as a measure of viral titer. To transduce *tax *alleles, 2 × 10^5 ^HeLa cells were first plated in a 6-well plate similar as in [37]. They were then transduced with LV (m.o.i. = 2) the next day. After 24 h, the medium was removed and fresh DMEM containing puromycin (1 μg/ml, Sigma) was added. The selection medium and cell debris were removed 48 h after selection by 1× PBS washes, and replaced with fresh puromycin-free DMEM.

### Immunoblot analyses of cells transduced with different *tax *alleles

HeLa cells transduced with LV-Tax or LV-mutant Tax were grown to approximately 70% confluency 4–5 days after initial seeding. SDS sample buffer (2×, 60 μl) was added to each well to lyse the cells. Cell lysates were scraped and transferred to an Eppendorf tubes and heated at 100°C for 5 minutes. Total cell proteins were then resolved by SDS/12% PAGE, transferred to nitrocellulose membrane and probed with antibodies (Santa Cruz Biotechnology) against cyclin B1 (sc-752), actin (sc-1616), I-κBα (sc-1643), p52-NFκB2 (sc-7386), Skp2 (sc-7164), p21^*CIP1*/*WAF1*^(sc-397), and p27^*KIP 1*^(sc-1641). For detection of Tax, a mouse hybridoma antibody, 4C5, which reacts with the COOH terminal region of Tax, was used.

### Cell cycle analysis of HeLa cells transduced with *tax *alleles

HeLa cells transduced with LV containing either the wild-type or each of the mutants *tax *alleles were selected with puromycin and maintained as described above. Cells were harvested 4–5 days post-transduction for flow cytometry as previously described [36].

### Detection of Clb2p in *S. cerevisia*

Detection of Clb2p in *S. cerevisia *was previously described [30] except that yeast cracking buffer (8M urea, 5% SDS, Tris-HCl (pH6.8), EDTA 0.1 mM, Bromophenol blue 0.4 mg/ml, β-mercaptoethanol 10 μl/ml) was used as the lysis buffer. Immunoblots were carried out with anti-HA (Santa Cruz Biotechnology), anti-PP2A-C (Upstate) and 4C5 (Tax) monoclonal antibodies.

### Detection of polyubiquitinated cyclin B

HEK 293T cells were co-transfected with HA-tagged ubiquitin and the various *tax *alleles as above. Cells are washed the next day and harvested 48 h later for immunoprecipitation using the cyclin B1 antibody (Santa Cruz Biotechnology) as previously described [36] except that RIPA buffer (Upstate protocol Tris-HCl, NP40 1%, Na-deoxycholate 0.25%, NaCl 150 mM, EDTA 1 mM, PMSF 1 mM, protease inhibitor cocktail (1 μg/ml), Na_3_VO_4 _1 mM, and NaF 1 mM) was used instead of the lysis buffer previously described. Immunoblots were carried out with anti-HA (Santa Cruz), anti-cyclin B1 (Santa Cruz Biotechnology) and 4C5 (Tax) monoclonal antibodies.

## Authors' contributions

RM and C-ZG were responsible for the design of the study and the draft of the manuscript. RM performed most of the experiments. CC isolated the *tax *mutants and assisted in the reporter assays. SH assisted in the analyses of *S. cerevisiae*-derived *tax* mutants. LZ and ML contributed the 18x21-EGFP reporter construct. YLK assisted in the cell cycle analyses. All authors read and approved the final manuscript.
